# Physicochemical and Mechanical Performance of Dental Resins Formulated from Dimethacrylated Oligoesters Derived from PET Recycling via Glycolysis

**DOI:** 10.3390/polym17192660

**Published:** 2025-10-01

**Authors:** Stefanos Karkanis, Alexandros K. Nikolaidis, Elisabeth A. Koulaouzidou, Dimitris S. Achilias

**Affiliations:** 1 Laboratory of Polymer and Color Chemistry and Technology, Department of Chemistry, Aristotle University Thessaloniki, 541 24 Thessaloniki, Greece; karkaniss@chem.auth.gr; 2 Division of Dental Tissues’ Pathology and Therapeutics (Basic Dental Sciences, Endodontology and Operative Dentistry), School of Dentistry, Aristotle University Thessaloniki, 541 24 Thessaloniki, Greece; nikolchem@dent.auth.gr (A.K.N.); koulaouz@dent.auth.gr (E.A.K.)

**Keywords:** dimethacrylate-based dental resins, alternative monomers, bisphenol A release, water sorption, mechanical properties

## Abstract

Growing concerns over the toxicity and sustainability of dental materials have driven the search for alternatives to bisphenol A-glycidyl methacrylate (Bis-GMA), a widely used dental resin monomer associated with health risks. This study highlights the potential of less health-hazardous dental formulations by incorporating high-value materials derived from the glycolysis of poly(ethylene terephthalate) (PET). Dimethacrylated oligoesters (PET-GLY-DM), synthesized through the methacrylation of PET glycolysis products, were blended with Bis-GMA and triethylene glycol dimethacrylate (TEGDMA), toward the gradual replacement of Bis-GMA content. The innovative PET-GLY-DM-based resins exhibited a higher degree of conversion compared to traditional Bis-GMA/TEGDMA formulations, as measured by FTIR spectroscopy, accompanied by an increase in polymerization shrinkage, evaluated via a linear variable displacement transducer system. While the incorporation of PET-GLY-DM slightly reduced flexural strength and elastic modulus, it significantly decreased water sorption, resulting in a smaller reduction in mechanical properties after water immersion for 7 days at 37 °C and improved long-term performance. Furthermore, PET-GLY-DM resins exhibited low bisphenol-A (BPA) release measured with HPLC. It was thus confirmed that PET-GLY-DM resins derived from the glycolysis of PET wastes represent a promising alternative to conventional light-cured dental resins, offering reduced BPA release and improved water resistance.

## 1. Introduction

Resin composites, widely used for tooth-colored dental restorations, primarily consist of methacrylic monomers and additives such as fillers, polymerization initiators and inhibitors [1]. Inorganic fillers typically constitute the largest weight fraction followed by methacrylic monomers, thus making up the bulk of the composite resin’s weight [2]. The methacrylic monomers act as binders for the fillers, significantly affecting the properties and performance of the resin composite [3,4]. Polyfunctional methacrylates are often included in monomer compositions due to their ability to enhance mechanical performance. Typically, these polyfunctional methacrylate compositions are based on bisphenol A glycidyl methacrylate (Bis-GMA), which is the most commonly used monomer in dental composites, pit and fissure sealants, and resin cements. Bis-GMA the diester of methacrylic acid and bisphenol A diglycidyl ether [5], contains two polymerizable groups, rendering it suitable for forming crosslinked polymers in dental applications. However, Bis-GMA monomer is usually accompanied with bisphenol A (BPA) traces. BPA is known to act as endocrine disruptor [6,7], raising concerns about its safe use. Short-term administration of Bis-GMA in animals or cell cultures can induce changes in estrogen-sensitive organs or cells [8]. Additionally, Bis-GMA is highly viscous, necessitating the inclusion of a low-viscosity monomer like triethylene glycol dimethacrylate (TEGDMA) so as to improve the workability of dental composite formulations [9,10]. However, incorporating excess TEGDMA leads to increased polymerization shrinkage and water sorption, compromising the ultimate mechanical properties [10].

Numerous efforts have been made to identify alternative monomers to replace Bis-GMA, with particular focus on urethane dimethacrylate (UDMA) derivatives [11,12,13]. These monomers are highly regarded for their enhanced flexibility, improved degree of conversion and lower polymerization shrinkage, making them excellent candidates for dental composite applications. Another promising group of alternatives consists of bio-based methacrylates derived from renewable resources, such as itaconates [14,15] and isosorbide methacrylates [16,17,18]. These sustainable monomers not only exhibit mechanical properties comparable to those of Bis-GMA systems, but also offer advantages like biocompatibility and low toxicity, making them well-suited for dental applications. In addition to these, other innovative options have been explored, including styrenic-methacrylates [19], vinyl cyclopropanes [20] and fluorinated methacrylates [21]. Each of these alternatives presents unique properties and benefits, solidifying their potential as replacements for Bis-GMA in advanced dental materials. More recently, other strategies have been investigated to further enhance the performance of BPA-free resin systems. For instance, the incorporation of biobased trimethacrylates has shown to improve both the physicochemical and mechanical properties of experimental resin composites, underscoring the potential of multifunctional renewable monomers to reinforce network structures [22]. Similarly, the adjustment of Bis-GMA/UDMA proportions in experimental formulations has been systematically studied, providing valuable insights into optimizing mechanical performance while maintaining favorable handling properties [23]. Moreover, the design of BPA-free dental resin composites with integrated antibacterial properties has emerged as a particularly promising direction, broadening their clinical applicability [24].

Our previous work [25] explored the potential usage of dimethacrylated oligoesters (PET-GLY-DMs), derived from chemical recycling of poly(ethylene terephthalate) (PET) bottles as photopolymerizable substitutes for Bis-GMA in dental resins. In this framework, PET was initially glycolized using specific PET/diethylene glycol (DEG) molar ratios, resulting in oligoester diols with molecular weights comparable to Bis-GMA. The gained oligomers were then methacrylated to form PET-GLY-DMs, which were proven to eluate no BPA species under extreme leaching conditions. Their further incorporation into dental resin formulations showed that the manipulation of PET-GLY-DMs portion into the light-curable monomer blends might lead to the lower viscosity of dimethacrylates’ mixtures and eventually to dental resins with enhanced degree of conversion in comparison to typical BisGMA/TEGDMA resins.

The present study focuses on a holistic approach to the investigation of physicochemical and mechanical attitude of dental resins based on alternative PET-GLY-DMs monomers. In this context, mixtures of PET-GLY-DMs/Bis-GMA/TEGDMA containing different amounts of PET-GLY-DMs were initially prepared in order to produce light-curable dental resins. Taking into account that the proposed PET-GLY-DMs are more hydrophobic than Bis-GMA and free of BPA impurities, it is expected that the replacement of Bis-GMA content with PET-GLY-DMs would minimize both the extent of BPA release and water sorption and solubility phenomena, which are closely related to the conventional dimethacrylate-based dental resins.

## 2. Materials and Methods

### 2.1. Materials

Τriethylene glycol dimethacrylate (TEGDMA) with a purity of 95% and 2,2-Bis[p-(2′-hydroxy-3′-methacryloxypropoxy)phenylene]propane (Bis-GMA, 99%) were obtained from Sigma-Aldrich (Steinheim, Germany) and used without further purification. The co-initiator 2-(dimethylamino)ethyl methacrylate (DMAEMA, 99%) and initiator camphorquinone (CQ, 98%), were obtained from J&K Scientific GmbH (Pforzheim, Germany) and used without further purification. Tetrahydrofuran (THF, 99.9%), used as a solvent in gel permeation chromatography (GPC), acetonitrile (CH_3_CN) CHROMASOLV^TM^ Gradient for HPLC (grade ≥ 99.9%) and water (H_2_O) CHROMASOLV^TM^Plus for HPLC were supplied from Honeywell-Riedel de Haen (Honeywell, Charlotte, NC, USA), while methanol (CH_3_OH) HPLC gradient grade (99.9%) was procured from ChemLab (Zedelgem, Belgium).

### 2.2. Preparation of Light-Curable Dental Resin Formulations

**Figure 1:**
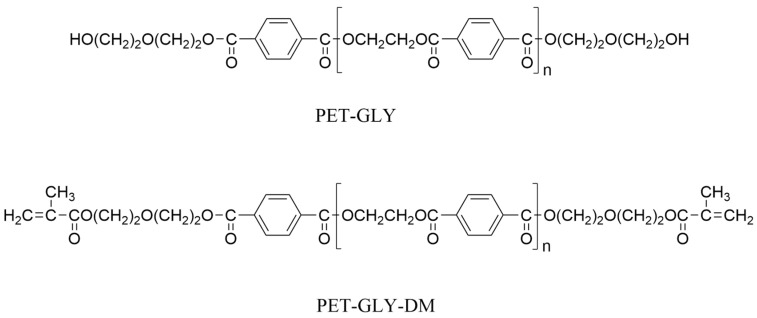
Chemical structures of PET-GLY and PET-GLY-DM.

The synthesis procedure of the starting dimethacrylate-based monomers, derived through the glycolysis of PET bottles, was fully described in our previous work [25]. Briefly, the depolymerization of PET was conducted using two PET/DEG molar ratios of 1:4 and 1:7, yielding oligoester diols of different molecular weights (PET-GLY 1 and PET-GLY 2). The hydroxyl groups of the PET-GLY products were subsequently converted into methacrylate groups via a methacrylation reaction, resulting indifferent dimethacrylated oligoesters (PET-GLY 1-DM and PET-GLY 2-DM). PET-GLY and PET-GLY-DM structures are shown in Figure 1, where n equals to 0, 1 and 2. Afterward, the two polymerizable PET-GLY-DMs were blended with Bis-GMA and TEGDMA to form light-curable dental resins of diverse compositions. The specific monomer ratios used for the preparation of the studied dental resins are presented in Table 1. A photoinitiating system consisted of CQ (0.2% *w*/*w*) and DMAEMA (0.8% *w*/*w*) was incorporated in each experimental formulation. In order to facilitate monomer mixing, the PET-GLY-DMs were heated up to 80–90 °C on a heating plate. Keeping light protection, TEGDMA and the photoinitiating system were then added under continuous stirring and heating at 90 °C for one hour until a homogenous mixture was achieved. The above-mentioned blend was then added to Bis-GMA, while simultaneous stirring and heating at 90 °C continued in an ultrasonic bath until a homogeneous mixture was finally obtained.

### 2.3. Degree of Conversion

Polymerization kinetics was assessed by placing a small amount of each experimental dimethacrylate-based monomer system between two translucent Mylar strips which were pressed between two glass plates to form a very thin film. The obtained films were exposed to visible light at different curing time intervals (5, 10, 20, 30, 40, 60, 80, 120, 180 s) using a LED light-curing device (Bluephase^®^ Style M8, Ivoclar Vivadent AG, Schaan, Liechtenstein) with an intensity of 800 mW·cm^−2^ ± 10%. They were immediately scanned using an attenuated total reflection Fourier transform infrared (ATR-FTIR) spectrometer (Cary 630, Agilent Technologies, Mulgrave, VIC, Australia). Samples were placed in a diamond. Spectral acquisition was performed over the 4000–600 cm^−1^ region. The resolution of the equipment was 4 cm^−1^, and the number of scans was 32. Spectra of the unset monomer blends were also obtained. Agilent MicroLab software (version 4.6) was used to manipulate the raw data of spectra. The area of aliphatic C=C peak absorption of 1637 cm^−1^ and the aromatic C=C peak absorption of 1608 cm^−1^ were determined utilizing a baseline technique, which proved to be the best fit according to the Beer–Lambert law [26]. The aromatic C=C vibration was used as an internal standard. After a total of five specimen repetitions (n = 5), the percent degree of monomer conversion (*DC*%) of the cured specimens, namely the percent amount of double carbon bond reacted at each time period, was determined according to Equation (1):

(1) $$DC\left(\%\right)=\left[1-\frac{{\left(A_{1637}/A_{1608}\right)}_{\mathrm{polymer}}}{{\left(A_{1637}/A_{1608}\right)}_{\mathrm{monomer}}}\right]\times 100$$

### 2.4. Polymerization Shrinkage

Polymerization shrinkage kinetics were assessed based on the “bonded-disk” method as described and later improved by Watts and co-workers [27,28,29]. To put it briefly, a disk-shaped unset specimen of 1.0 mm by 8.0 mm (height by diameter) was created and positioned in the center of a hard glass plate that was 3 mm thick. To guarantee adhesion, a flexible cover-slip diaphragm with an internal diameter of about 15 mm was placed on top of the specimen disk and held up by an outside peripheral brass ring. On the cover slip, a uniaxial linear variable displacement transducer (LVDT) measurement device was positioned in the center. A transducer indication (E 309, RDP Electronics Ltd., Wolverhampton, UK) and a high-resolution analog-to-digital converter (ADAM-4016 acquisition module) were used to send the signal from the LVDT to a computer. Data acquisition was facilitated by the datalogger software AdvantechAdam/Apax.NET Utility, version 2.05.11. Measurements were recorded by continuously irradiating the specimens with the aforementioned LED polymerization unit for 5 min from beneath the glass plate at room temperature. Five repetitions (n = 5) were conducted for each specimen. Strain was calculated according to Equation (2):

(2) $$\varepsilon (\%)=(\Delta L/L_{0})\times 100$$

where *ε* (%) represents the strain (%) and Δ*L* and *L*_0_ are the shrinkage displacement and the initial specimen thickness, respectively.

### 2.5. Water Sorption and Solubility

The water sorption and solubility parameters were evaluated following the procedure outlined in ISO 4049-2019 [30]. To do this, four specimen disks (n = 4) with a diameter of 15 mm and a thickness of 1 mm were fabricated for each experimental resin by filling a Teflon mold with the unpolymerized mixture, as previously outlined. The samples were irradiated for 40 s on each side by partitioning them into nine overlapping irradiation zones with the aforementioned LED polymerization device (Bluephase^®^ Style M8, Ivoclar Vivadent AG, Schaan, Liechtenstein). All specimens were positioned in a desiccator and subsequently moved to a pre-conditioning oven maintained at 37 ± 1 °C. They were taken out after 24 h, kept for two h at 23 ± 1 °C in a second desiccator and weighed using a Sartorius TE 124S balance (Sartorius AG, Göttingen, Germany) to within ± 0.1 mg. Until a constant mass (*m*_1_) was achieved, this cycle was repeated. To determine the volume (*V*), the average thickness and diameter of each specimen were measured using a digital micrometer with an accuracy of ± 0.02 mm. After that, the disks spent seven days submerged in water at 37 ± 1 °C. They were then taken out, blotted dry to get rid of more moisture, waved for 15 s and weighed (*m*_2_). Following the previously mentioned cycle, the specimens were subsequently reconditioned to a constant mass (*m*_3_) in the desiccators.

The following Equation (3) was employed to estimate the water sorption, *W_sp_* (μg mm^−3^):

(3) $$W_{sp}=\frac{m_{2}-m_{3}}{V}$$

where *m*_2_ is the mass of the specimen (μg) after immersion in water for 7 days, *m*_3_ is the mass of the reconditioned specimen (μg) and *V* is the volume of the specimen (mm^3^).

The values for solubility, *W_sl_* (μg mm^−3^), were calculated from Equation (4):

(4) $$W_{sl}=\frac{m_{1}-m_{3}}{V}$$

where *m*_1_ is the conditioned mass (μg) of the specimen prior to immersion in water.

### 2.6. Measurement of Mechanical Properties

For flexural tests, bar-specimens were prepared by filling a Teflon mold (2 mm × 2 mm × 25 mm) with unpolymerized mixture in accordance with ISO 4049-2019 [30]. The mold was then irradiated from both sides using the above LED polymerization unit, with each side receiving overlapping exposures for 40 s. Five specimen bars (n = 5) were prepared for each experimental resin and stored at 37 °C under dark conditions for 24 h. The specimens were then immersed in water for 7 days at 37 ± 1 °C. Afterwards, the bending tests were performed using a 3-point transverse testing rig with a 20 mm span between the supports. This rig was mounted on a universal testing machine (Shimadzu EZ Flexural Tester, Model EZ-LX, Kyoto, Japan) with a 2 kN load cell. The bending process was carried out at a crosshead speed of 0.5 mm/min until the specimens fractured. During the tests, both the load and the corresponding deflection were recorded. The data were subsequently analyzed using the Trapezium of Shimadzu Version 1.5.6 software. The calculations for flexural strength (*σ*) in MPa and elastic modulus (*E*) in GPa were calculated according to Equations (5) and (6):

(5) $$\sigma =\frac{3F_{max}\cdot l}{2bh^{2}}$$

(6) $$Ε=\frac{F_{1}l^{3}}{4bd_{1}h^{3}}\times 10^{-3}$$

where *F*_1_ is the load recorded in N, *F_max_* is the maximum load recorded before fracture in N, *l* is the span between the supports (20 mm), *b* is the width of the specimen in mm, *h* is the height of the specimen in mm and *d*_1_ is the deflection in mm corresponding to the load *F_1_.*

### 2.7. Analysis of Organic Eluates from Dental Resins

#### 2.7.1. Sample Preparation

Uncured monomer blends were poured into Teflon (PTFE) molds to create disks that were 5 mm in diameter and 2 mm thick [30]. The mold surfaces were layered with glass slides covered with a Mylar sheet to eliminate air entrapping and adherence of the final set resin. The assembly was irradiated and secured with spring clips. A curing LED light (Bluephase^®^ Style M8, Ivoclar Vivadent AG, Schaan, Liechtenstein) with an intensity of 800 mW·cm^−2^ ± 10% was used to polymerize the disks. All disks were irradiated on both sides for 40 s. Four disks (n = 4) were prepared for each studied resin. The disks were detached from the molds and then immersed in 8 mL amber glass vials containing 1 mL methanol. The vials were sealed with polypropylene caps internally coated with PTFE septa to prevent evaporation and were kept in an incubator at 37 °C for 3 weeks. Each methanol eluate was then filtered through a PTFE filter disk (0.22 μm) and transferred to separate HPLC vials in order to be injected into the autosampler of the liquid chromatography system.

#### 2.7.2. Liquid Chromatography Analysis

A high-pressure liquid chromatography system (HPLC) (Shimadzu LC-2050C, Shimadzu Corp., Kyoto, Japan) equipped with a C18 column (Shim-pack GIST, Shimadzu Corp, 150 × 4.6 mm, 5 μm) was used for the separation and determination of the targeted compounds. The mobile phase was a mixture of acetonitrile and water (CH_3_CN:H_2_O, 70:30% *v*/*v*) delivered isocratically with a flow rate of 1 mL/min. The injection volume of each sample was 25 μL, and the column’s temperature was set at 37 ± 0.1 °C. The detection wavelength of the photodiode array detector was adjusted at 203 nm. Bis-GMA, TEGDMA and BPA methanol stock solutions of 500 μg/mL were initially prepared and subsequently diluted in order to obtain the suitable working range concentrations. Qualification of analytes was based on the comparison of their retention times to those of the corresponding prepared methanol standards. Calibration curves were plotted as regression lines (*y* = bx + a) for each analyte separately by analyzing the diluted working solutions. Specifically for BPA, the equation was y = 16,195x−17,955 with a correlation coefficient (R^2^) of 0.999. For TEGDMA, the equation was y = 95,485x + 36,340 with an R^2^ of 0.999, while for Bis-GMA, the equation was y = 16,311x + 37,008 with an R^2^ of 0.999. The limit of detection (LOD) and limit of quantitation (LOQ) were determined as 5.36 and 16.25 μg/mL for BPA, 0.86 and 2.60 μg/mL for Bis-GMA, as well as 2.23 and 6.75 μg/mL for TEGDMA, respectively.

### 2.8. Statistical Analysis

The assumption of normal distribution was investigated for the variables using the Shapiro–Wilk test. Degree of conversion, polymerization shrinkage, water sorption and solubility, flexural strength, elastic modulus and BPA, TEGDMA, Bis-GMA concentrations for six groups were analyzed by one-way analysis of variance (ANOVA) and Tukey’s test. Statistical analysis was performed using Origin Pro software (version 7.5). Statistical significance was accepted at *p* ≤ 0.05.

## 3. Results

### 3.1. Evaluation of Polymerization Kinetics

**Figure 2:**
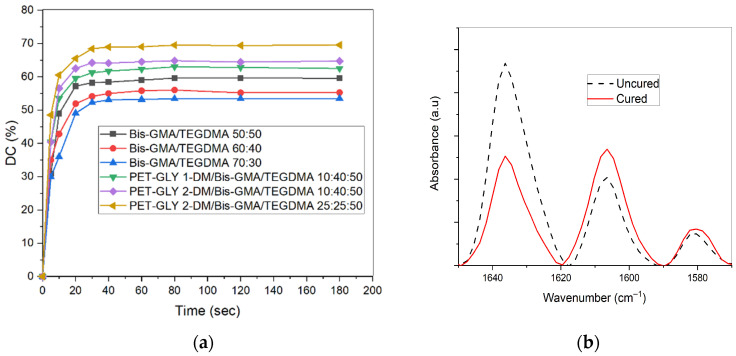
(**a**) Plots illustrating the degree of conversion (DC%) versus curing time for the experimental dimethacrylate-based dental resins and (**b**) FT-IR spectrum with measured peak areas (1637 and 1608 cm^−1^) used to calculate the percent degree of conversion (DC%) for uncured and cured experimental resins.

The degree of conversion (*DC*) of the experimental dental resins was monitored over time (Figure 2a), while the final *DC* (%) values achieved for the tested experimental resins after 180 s light-curing are provided in Table 2. An example of FT-IR plot for both uncured and cured experimental resin, which was used to calculate monomer conversion, is shown in Figure 2b. As depicted in Figure 2a, the optimal curing time reflecting the maximum conversion was approximately 80 s for the tested resins, which reached conversion values in the range of 53.46–69.54%. The PET-GLY-DM-containing blends exhibited an increasing trend in DC values, with PET-GLY-DM/Bis-GMA/TEGDMA formulations achieving conversion rates of 62.5, 64.72 and 69.54%, compared to 53.46, 55.26 and 59.56% for Bis-GMA-only formulations. Additionally, the PET-GLY 2-DM-containing PET-GLY-DM/Bis-GMA/TEGDMA 10:40:50% *w*/*w* blend exhibited an increased *DC* in comparison with the respective PET-GLY 1-DM-containing blend. Non-statistically significant differences were observed between the Bis-GMA/TEGDMA 60–40% *w*/*w* and Bis-GMA/TEGDMA 70–30% *w*/*w* groups, as well as between PET-GLY 1-DM/Bis-GMA/TEGDMA (10/40/50% *w*/*w*) and PET-GLY 2-DM/Bis-GMA/TEGDMA (10/40/50% *w*/*w*) groups. Significant differences were observed between the remaining groups (*p* ≤ 0.05).

### 3.2. Determination of Polymerization Shrinkage Kinetics

**Figure 3:**
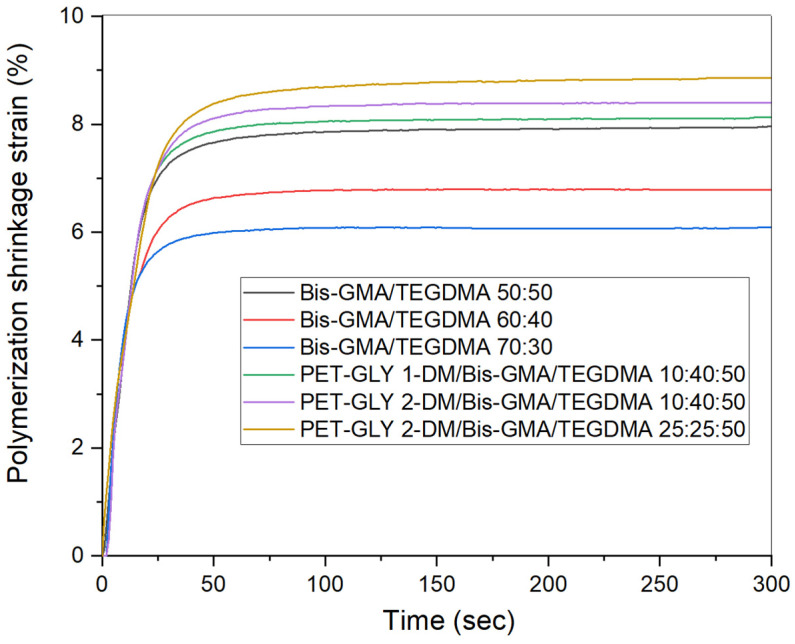
Time dependence of polymerization shrinkage strain of the synthesized dimethacrylate-based dental resins.

The polymerization shrinkage strain over time for the studied experimental resins is presented in Figure 3, whereas the ultimate strain values are provided in Table 2. The data listed in Table 2 reveal that the obtained strain values follow the increasing tendency of the degree of conversion. Figure 2 shows that the strain determined for the Bis-GMA/TEGDMA 50:50, 60:40 and 70:30% *w*/*w* blends ranged from approximately 6.09% to 7.96%, with the 50:50 blend demonstrating the highest shrinkage. Resins enriched with PET-GLY-DM monomers exhibited higher-shrinkage strains, reaching up to 8.85%. Comparing the blends of PET-GLY-DM/Bis-GMA/TEGDMA 10:40:50% *w*/*w*, the particular mixture containing PET-GLY 2-DM exhibited greater shrinkage. Non-statistically significant differences were observed between the Bis-GMA/TEGDMA (50–50% *w*/*w*), PET-GLY 1-DM/Bis-GMA/TEGDMA (10/40/50% *w*/*w*) and PET-GLY 2-DM/Bis-GMA/TEGDMA (10/40/50% *w*/*w*) groups, as well as between PET-GLY 2-DM/Bis-GMA/TEGDMA (10/40/50% *w*/*w*) and PET-GLY 2-DM/Bis-GMA/TEGDMA (25/25/50% *w*/*w*) groups. Significant differences were observed between the remaining groups (*p* ≤ 0.05).

### 3.3. Water Sorption and Solubility Results

**Figure 4:**
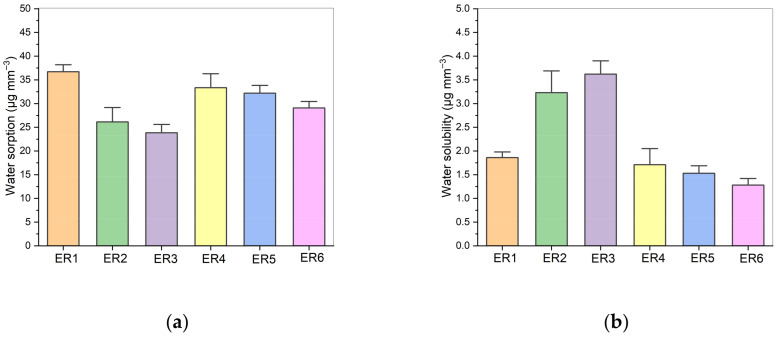
(**a**) Water sorption plots and (**b**) water solubility plots of the tested experimental resins.

The water sorption and solubility values of the experimental resins are given in Table 2. Representative bar charts reflecting the observed tendencies are also depicted in Figure 4. When the TEGDMA content was set at 50%, the introduction of PET-GLY-DM into the blend decreased water sorption compared to the Bis-GMA/TEGDMA 50:50 blend. Concerning the two PET-GLY-DM/Bis-GMA/TEGDMA blends, the incorporation of the highest-molecular-weight PET-GLY 1-DM monomer slightly increased water sorption. However, when the water sorption values for the PET-GLY-DM-containing blends (33.36 μg mm^−3^, 32.19 μg mm^−3^, 29.09 μg mm^−3^) were compared with the Bis-GMA/TEGDMA 60:40 (26.13 μg mm^−3^) and 70:30 (23.85 μg mm^−3^) controls, an increase in water sorption was observed compared to these controls. Non-statistically significant differences were observed between the Bis-GMA/TEGDMA (50–50% *w*/*w*) and PET-GLY 1-DM/Bis-GMA/TEGDMA (10/40/50% *w*/*w*) groups, as well as between Bis-GMA/TEGDMA (60/40% *w*/*w*), Bis-GMA/TEGDMA (70/30% *w*/*w*) and PET-GLY 2-DM/Bis-GMA/TEGDMA (25/25/50% *w*/*w*) groups. Non-statistically significant differences were also observed between PET-GLY 1-DM/Bis-GMA/TEGDMA (10/40/50% *w*/*w*) and PET-GLY 2-DM/Bis-GMA/TEGDMA (10/40/50% *w*/*w*) groups. Significant differences were observed between the remaining groups (*p* ≤ 0.05).

The solubility results reflecting the amount of residual monomer extracted by water after 7 days aging at 37 °C are also presented in Table 2. Bis-GMA/TEGDMA networks, which exhibited a lower degree of conversion compared to PET-GLY-DM/Bis-GMA/TEGDMA counterparts, showed higher solubility. Notably, the Bis-GMA/TEGDMA 70:30% *w*/*w* system yielding the lowest DC exhibited the highest solubility in water (3.62 μg mm^−3^). In contrast, the PET-GLY 2-DM/Bis-GMA/TEGDMA 25:25:50% *w*/*w* resin, having the highest DC value, demonstrated the lowest solubility (1.28 μg mm^−3^). Non-statistically significant differences were observed between the Bis-GMA/TEGDMA (50–50% *w*/*w*), PET-GLY 1-DM/Bis-GMA/TEGDMA (10/40/50% *w*/*w*), PET-GLY 2-DM/Bis-GMA/TEGDMA (10/40/50% *w*/*w*) and PET-GLY 2-DM/Bis-GMA/TEGDMA (25/25/50% *w*/*w*) groups, as well as between Bis-GMA/TEGDMA (60/40% *w*/*w*) and Bis-GMA/TEGDMA (70/30% *w*/*w*) groups. Significant differences were observed between the remaining groups (*p* ≤ 0.05).

### 3.4. Mechanical Properties

**Figure 5:**
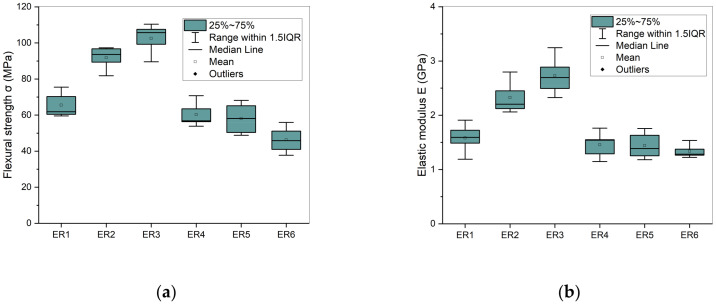
Box plots drawn for (**a**) flexural strength and (**b**) elastic modulus of the experimental resins after immersion in distilled water for 1 week.

**Figure 6:**
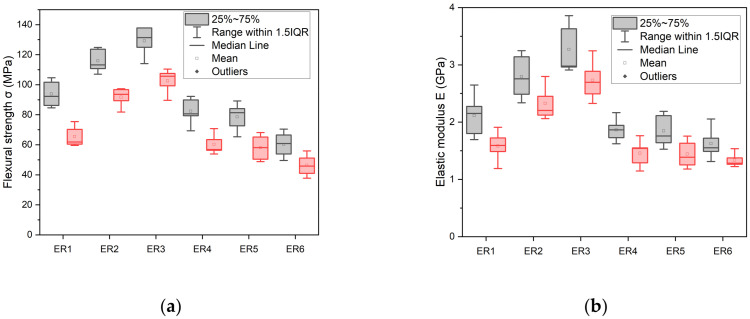
Box plots drawn for (**a**) flexural strength and (**b**) elastic modulus of the experimental resins before 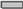 [25] and after 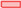 immersion in distilled water for 1 week.

The comparative charts illustrating the flexural properties of the synthesized dental resins after one week storage in water are represented in Figure 5. The calculated mean values for flexural strength (FS) and elastic modulus (EM), accompanied by their standard deviations (SD), are detailed in Table 3. For comparison reasons, data referring to flexural response of the same dimethacrylate-based resins prior to their immersion in water, as they were described in our previous work [25] , are also included in Table 3, as well as in Figure 6. Table 3 demonstrates that the Bis-GMA/TEGDMA 50:50 sample showed the highest reduction in both flexural strength (from 93.89 to 65.53 MPa, 30.21%) and elastic modulus (from 2.11 to 1.58 GPa, 25.12%) after one week of aging, as this resin exhibited the highest water sorption value (36.72 μg mm^−3^). Keeping the percentage of TEGDMA constant at 50% in specific experimental resins, the introduction of dimethacrylated oligoesters decreased hydrophilicity and water sorption, and therefore resulted in a smaller decline in mechanical properties. The reduction in their FS and EM values was 26.76% and 22.04% for ER4, 25.97% and 21.74% for ER5 and 23.09% and 17.90% for ER6, respectively. However, regarding the Bis-GMA/TEGDMA resins characterized by lower TEGDMA percentages (60:40, 70:30), the reduction in their FS and EM values was the lowest (60:40, 20.80% and 16.49%, respectively), (70:30, 14.02% and 16.26%, respectively) and clearly correlated with their lowest water sorption values (26.13 μg mm^−3^ and 23.85 μg mm^−3^, respectively).

Regarding FS, non-statistically significant differences were observed between the Bis-GMA/TEGDMA (50–50% *w*/*w*), PET-GLY 1-DM/Bis-GMA/TEGDMA (10/40/50% *w*/*w*) and PET-GLY 2-DM/Bis-GMA/TEGDMA (10/40/50% *w*/*w*) groups, as well as between Bis-GMA/TEGDMA (60/40% *w*/*w*) and Bis-GMA/TEGDMA (70/30% *w*/*w*) groups. Non-statistically significant differences were also observed between PET-GLY 1-DM/Bis-GMA/TEGDMA (10/40/50% *w*/*w*), PET-GLY 2-DM/Bis-GMA/TEGDMA (10/40/50% *w*/*w*) and PET-GLY 2-DM/Bis-GMA/TEGDMA (25/25/50% *w*/*w*) groups. Significant differences were observed between the remaining groups (*p* ≤ 0.05). In terms of EM, non-statistically significant differences were observed between the Bis-GMA/TEGDMA (50–50% *w*/*w*), PET-GLY 1-DM/Bis-GMA/TEGDMA (10/40/50% *w*/*w*), PET-GLY 2-DM/Bis-GMA/TEGDMA (10/40/50% *w*/*w*) and PET-GLY 2-DM/Bis-GMA/TEGDMA (25/25/50% *w*/*w*) groups, as well as between Bis-GMA/TEGDMA (60/40% *w*/*w*) and Bis-GMA/TEGDMA (70/30% *w*/*w*) groups. Non-statistically significant differences were also observed between PET-GLY 1-DM/Bis-GMA/TEGDMA (10/40/50% *w*/*w*), PET-GLY 2-DM/Bis-GMA/TEGDMA (10/40/50% *w*/*w*) and PET-GLY 2-DM/Bis-GMA/TEGDMA (25/25/50% *w*/*w*) groups. Significant differences were observed between the remaining groups (*p* ≤ 0.05).

Regarding FS and EM before and after immersion, in terms of FS, significant difference was observed between PET-GLY 2-DM/Bis-GMA/TEGDMA (25/25/50% *w*/*w*) (*p* ≤ 0.05) and non-statistically significant differences were observed between the remaining groups. Regarding EM, non-statistically significant differences were observed between Bis-GMA/TEGDMA (50–50% *w*/*w*), Bis-GMA/TEGDMA (60–40% *w*/*w*) and PET-GLY 1-DM/Bis-GMA/TEGDMA (10/40/50% *w*/*w*). Significant differences were observed between the remaining groups (*p* ≤ 0.05).

### 3.5. Detection and Quantification of Resins’ Organic Eluates

**Figure 7:**
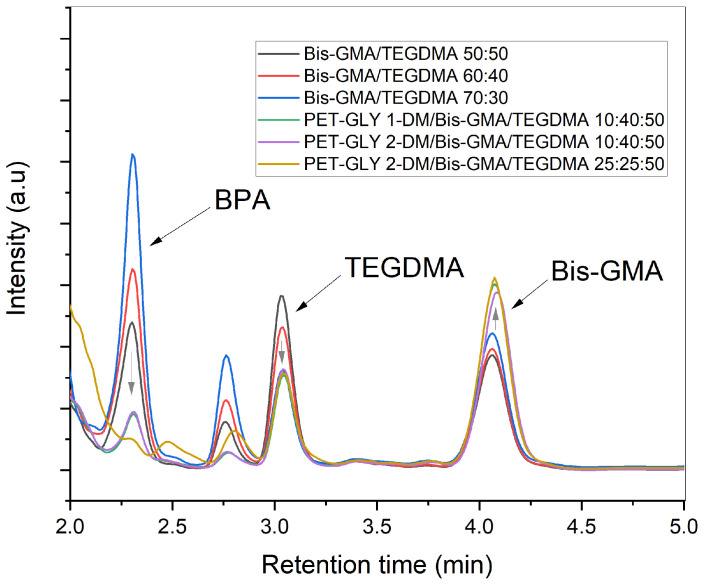
HPLC chromatogram of each experimental resin’s methanolic extract.

Figure 7 illustrates the HPLC chromatograms acquired for methanolic extracts from different formulated dental resins. Additionally, the quantified concentrations of eluted BPA, TEGDMA and Bis-GMA are listed in Table 4. The peak recorded at 2.3 min was found to identify BPA, the peak at 3.04 min corresponded to TEGDMA, while the peak at 4.09 min was indicative of Bis-GMA detection. The targeted retention times were confirmed based on the chromatographic profile of each individual monomer standard. Regarding the Bis-GMA/TEGDMA systems, the peak intensity of Bis-GMA increased as the proportion of Bis-GMA in the formulation rose. Accordingly, the Bis-GMA/TEGDMA 70:30 resin released the maximum amount of Bis-GMA, namely 0.246 mg Bis-GMA/mg resin. Conversely, the chromatogram peak corresponding to TEGDMA attenuated as the proportion of Bis-GMA in the resin composition increased. In particular, the leached amount of TEGDMA decreased from 0.038 to 0.015 mg TEGDMA/mg resin when the Bis-GMA/TEGDMA ratio was altered from 50:50 to 70:30 *w*/*w*. In contrast, the introduction of PET-GLY-DM in the PET-GLY-DM/Bis-GMA/TEGDMA samples resulted in a significant reduction in BPA detection compared to the neat Bis-GMA/TEGDMA resins. Specifically, BPA traces were undetectable in the PET-GLY 2-DM/Bis-GMA/TEGDMA 25:25:50 sample. The amount of BPA eluted from PET-GLY-DM-modified resins (0.028 mg BPA/mg resin) was significantly lower than the BPA released from Bis-GMA/TEGDMA (0.124–0.357 mg BPA/mg resin). Regarding the chromatograms of PET-GLY-DM/Bis-GMA/TEGDMA eluates, the peak at 3.04 min for TEGDMA was weaker compared to the control Bis-GMA/TEGDMA resins, with TEGDMA release ranging from 0.015 to 0.017 mg TEGDMA/mg resin versus the 0.017–0.038 mg TEGDMA/mg resin, respectively. Furthermore, the Bis-GMA peak at 4.03 min was more pronounced in the PET-GLY-DM/Bis-GMA/TEGDMA extracts (0.273–0.290 mg Bis-GMA/mg resin), despite the lower Bis-GMA content in the resin composition. Regarding BPA concentration, non-statistically significant differences were observed between PET-GLY 1-DM/Bis-GMA/TEGDMA (10/40/50% *w*/*w*), PET-GLY 2-DM/Bis-GMA/TEGDMA (10/40/50% *w*/*w*) and PET-GLY 2-DM/Bis-GMA/TEGDMA (25/25/50% *w*/*w*) groups. Significant differences were observed between the remaining groups (*p* ≤ 0.05). In terms of TEGDMA concentration, non-statistically significant differences were observed between PET-GLY 1-DM/Bis-GMA/TEGDMA (10/40/50% *w*/*w*) and PET-GLY 2-DM/Bis-GMA/TEGDMA (10/40/50% *w*/*w*) groups, as well as between Bis-GMA/TEGDMA 70/30% *w*/*w* and PET-GLY 2-DM/Bis-GMA/TEGDMA (25/25/50% *w*/*w*) groups. Significant differences were observed between the remaining groups (*p* ≤ 0.05). Finally, regarding Bis-GMA concentration, non-statistically significant differences were observed between Bis-GMA/TEGDMA (50/50% *w*/*w*), Bis-GMA/TEGDMA (60/40% *w*/*w*) and Bis-GMA/TEGDMA (70/30% *w*/*w*) groups, as well as between the PET-GLY 1-DM/Bis-GMA/TEGDMA 10/40/50% *w*/*w*, PET-GLY 2-DM/Bis-GMA/TEGDMA 10/40/50% *w*/*w* and PET-GLY 2-DM/Bis-GMA/TEGDMA (25/25/50% *w*/*w*) groups.

## 4. Discussion

DC values were close to the literature data for Bis-GMA/TEGDMA-based resins ranging from 50% to 70% [3,31,32]. It is clear that an abrupt increase in the DC was achieved within a short period time due to the auto-acceleration or gel effect attributed to the influence of the diffusion-controlled phenomena on the termination reaction [33]. This initial surge was followed by a plateau in the DC curve, caused by the restriction of macroradical movement due to the developing polymer network. The growing viscosity of the formed resins through the glass effect, as controlled by the diffusion phenomena on the propagation reaction, ultimately set the calculated DC values [34,35,36]. High viscosity restricts the mobility of the molecular chains, hindering termination and propagation processes during photo-curing, leading to a decreased final conversion [37,38]. It is observed that the DC increased by rising TEGDMA content across all copolymer groups (Table 2), likely due to the lower medium viscosity and the specific configuration of the resin’s chemical structure, thus facilitating greater molecular mobility [31,39]. On the other hand, the lowest portion of TEGDMA in control Bis-GMA/TEGDMA 70:30% *w*/*w* blend did not favor further DC increment. Moreover, blends containing PET-GLY-DMs exhibited an increased trend in DC values possibly due to the introduction of alternative oligomers with ether groups, which were also proven to enhance monomer flexibility and, finally, to reduce viscosity against the control blends [25]. Therefore, it could be stated that, in the case of control samples, TEGDMA polymerization starts in a highly viscous medium, which accelerates the diffusion-controlled termination reaction to a higher extent when compared to less-viscous base monomer blends [40]. Furthermore, the presence of the PET-GLY 2-DM in the PET-GLY-DM/Bis-GMA/TEGDMA 10:40:50% *w*/*w* blend imparted an augmentation to the degree of conversion due to the reduced viscosity of monomer mixture [25]. The later finding discloses the potential influence of the PET/DEG molar ratio used for PET glycolysis in the ultimate DC of the obtained dimethacrylate-based resin.

The setting contraction of dental resins is the result of the conversion of longer intermolecular van der Waals distances of the resin monomers to shorter covalent bond lengths during light-curing [5,41], incorporating monomers like TEGDMA into the polymer network [37]. In dental practice, this process creates internal contraction stresses into the restorative material, leading to resin deformation in the surrounding tooth structure [38] and finally to gap formation at the tooth-resin interface [42,43]. When contraction forces exceed the bonding strength at the interface, the resulting gap can lead to staining, marginal leakage [44,45], post-operative sensitivity [46] and recurrent caries [47]. The obtained strain values follow the increasing tendency of the degree of conversion, which is in agreement with the literature data [48,49,50,51], as polymerization shrinkage is related to the percentage of the reacted double bonds. TEGDMA-rich matrices exhibit increased shrinkage due to an antiplasticizing effect, allowing for the closer packing of polymer chains [52]. Furthermore, higher Bis-GMA ratios resulted in lower shrinkage strains due to the lower double-bond conversion in a more restricted reaction environment [53,54]. In contrast, resins enriched with PET-GLY-DMs monomers exhibited higher shrinkage strains. It is apparent that the elevated DC of the resins containing dimethacrylated oligoesters may account for their stronger contraction compared to the Bis-GMA/TEGDMA controls. Comparing the blends of PET-GLY-DM/Bis-GMA/TEGDMA 10:40:50% *w*/*w*, the particular mixture containing the low-molecular-weight PET-GLY 2-DM exhibited greater shrinkage. This is consistent with a previous study mentioning that, when comparing monomers of the same functionality, polymerization shrinkage increases as monomer molecular weight decreases [5].

All experimental resins presented sorption values which conform to the sorption criteria of ISO 4049 (<40 µg mm^−3^) [30]. Water sorption of copolymers is influenced by both their hydrophilicity [55,56] and cross-link density [57,58,59]. According to Henry’s law and free volume theory, glassy polymers have a non-equilibrium liquid structure with an equilibrium hole-free volume that enables water diffusion. In cross-linked polymers like polydimethacrylates, the macromolecular packing density reduces hole-free volume, limiting solvent permeability and polymer chain swelling [60,61]. During the polymerization of multifunctional monomers, pendant double-bonds may undergo intramolecular reactions, forming primary cycles, secondary cycles (multiple cross-links), or cross-links [62]. While primary cycles increase conversion, they do not contribute to network formation [53] and generate microgels, creating heterogeneous networks with varying cross-linking densities, thus reducing the effective cross-linking density [63]. TEGDMA, being flexible, promotes pendant cyclization more than Bis-GMA [62], which is sterically hindered by its rigid aromatic rings. This higher cyclization in TEGDMA reduces effective cross-linking density, increases hole-free volume, and leads to greater water uptake and swelling. For this reason, the increase in TEGDMA percentage from 30% (23.85 μg mm^−3^) to 50% (36.72 μg mm^−3^) in pure Bis-GMA/TEGDMA systems favored the water sorption. Although the new alternative PET-GLY-DMs monomers contain alkyl segments characterized by the presence of ether groups, they, however, exhibit lower hydrophilicity than Bis-GMA, which has two hydroxyl groups capable of forming hydrogen bonds and thereby promoting a water-swelling effect [64]. Consequently, when the TEGDMA content was set at 50%, the introduction of PET-GLY-DM into the blend decreased water sorption compared to the Bis-GMA/TEGDMA 50:50 blend. Concerning the two PET-GLY-DM/Bis-GMA/TEGDMA blends, the incorporation of the highest-molecular-weight PET-GLY 1-DM monomer slightly increased water sorption, probably due to the increased ether group existence in the larger polymer chains. However, when the water sorption values for the PET-GLY-DM-containing blends (33.36 μg mm^−3^, 32.19 μg mm^−3^, 29.09 μg mm^−3^) were compared with the Bis-GMA/TEGDMA 60:40 (26.13 μg mm^−3^) and 70:30 (23.85 μg mm^−3^) controls, an increase in water sorption was observed compared to these controls. This suggests that the physical structure of the polymer network has a more significant impact on water sorption than the hydrophilicity of individual monomers, as resins with a lower TEGDMA percentage exhibited the lowest water sorption values.

All experimental resins showed solubility values in accordance with the solubility criteria of ISO 4049 (<7.5 µg mm^−3^) [30]. Bis-GMA/TEGDMA networks, which exhibited a lower degree of conversion compared to PET-GLY-DM/Bis-GMA/TEGDMA counterparts, showed higher solubility. This observation aligns with the principle that a higher degree of conversion, which typically results in a fewer unreacted monomers being integrated into the network, tends to lower the solubility of the polymer [31]. This is because the less unreacted monomer remains in a free state, reducing the potential for leaching. During the polymerization process, the unreacted monomer is entrapped inside the microvoids formed between the macromolecular chains and remains intact in the cross-linked network. Therefore, the solubility value is inversely related to the degree of polymer conversion and the distribution of unreacted monomers within the polymer matrix.

It is apparent that, regardless of the aging conditions, an increase in FS and EM parameters was observed when higher Bis-GMA percentage was applied in resin blends. The Bis-GMA monomer probably contributes to the increased hydrogen bonding, which might further enhance the cohesion and rigidity of the formed macromolecular chains, leading to the final stabilization of the polymer network. This result may also be due to the fact that an increased effective cross-linking density is observed with the increase in Bis-GMA and consequent decrease in TEGDMA, as TEGDMA leads to cyclizations in the cured resin. This increased effective cross-linking density enhances its mechanical strength [53]. Table 3 demonstrates that the Bis-GMA/TEGDMA 50:50 sample showed the highest reduction in both flexural strength and elastic modulus after one week of aging, as the above resin exhibited the highest water sorption value. This observation aligns with the finding that water molecules that are firmly bound to polar sites along the polymer networks induce swelling and plasticize the polymer, thus causing the reduction in the polymer’s mechanical properties by altering the mobility of their chain segments [65,66,67]. As demonstrated in Table 3, resins with lower water sorption values exhibited a smaller reduction in flexural strength (FS) and elastic modulus (EM) after one week of water storage. This correlation highlights the beneficial role of hydrophobic dimethacrylated oligoesters, which limit water uptake, thereby reducing the extent of polymer plasticization and hydrolytic degradation. The lower reduction in mechanical properties for these formulations may suggest enhanced structural stability, as water-induced swelling is minimized. Thus, the observed decrease in water sorption directly contributes to maintaining the mechanical integrity of the resins over time, indicating their potential for improved long-term performance.

A clear correlation was observed between the increasing proportion of Bis-GMA in the resin composition and the heightened levels of released BPA. Given that BPA is a synthetic precursor of Bis-GMA and may exist as a side product in the resin’s composition, higher concentrations of Bis-GMA resulted in greater amounts of leached BPA. Indeed, the maximum level of BPA released from the Bis-GMA/TEGDMA system was 0.357 mg BPA/mg resin when the monomer ratio was set at 70:30 *w*/*w*. In contrast, the introduction of PET-GLY-DM in the PET-GLY-DM/Bis-GMA/TEGDMA samples resulted in a significant reduction in BPA detection compared to the neat Bis-GMA/TEGDMA resins. In particular, BPA traces were undetectable for the PET-GLY 2-DM/Bis-GMA/TEGDMA 25:25:50 sample, which had the highest PET-GLY-DM content, thus demonstrating the effectiveness of PET-GLY-DM in mitigating BPA release. The increased detectability of Bis-GMA in PET-GLY-DM-containing systems, despite the lower Bis-GMA content, suggests that PET-GLY-DM competes with Bis-GMA during polymerization, inhibiting its reaction with TEGDMA. The greater flexibility of PET-GLY-DM likely enhances polymer chain mobility, favoring the reaction of PET-GLY-DM radicals with TEGDMA rather than Bis-GMA. Consequently, TEGDMA was consumed more effectively in PET-GLY-DM/Bis-GMA/TEGDMA samples, leading to fewer free molecules available for leaching. This is consistent with the observed higher degree of conversion in PET-GLY-DM-containing resins compared to Bis-GMA/TEGDMA controls, indicating the more efficient utilization of TEGDMA, a flexible molecule that contributes to an increased degree of conversion.

## 5. Conclusions

This study demonstrates the potential of eco-friendly dental resins formulated with dimethacrylated oligoesters derived from recycled PET. The novel PET-GLY-DM-containing resins exhibited a higher degree of conversion compared to standard Bis-GMA/TEGDMA resins, resulting in enhanced polymerization efficiency. Despite higher conversion, these resins maintained controlled polymerization shrinkage, reducing the risk of gap formation. Although the introduction of PET-GLY-DM reduced flexural strength and elastic modulus, it decreased water sorption, resulting in a smaller decline in mechanical properties after water immersion and enhancing long-term durability. Importantly, PET-GLY-DM-containing resins showed relatively limited levels of BPA, addressing the safety concerns associated with conventional light-cured dental resins. For future studies, comprehensive biocompatibility assessments, including cytotoxicity and cell viability tests, can be performed to further validate the safety of these materials. In addition, aspects related to scalability and industrial reproducibility could be investigated in economic feasibility studies to assess their potential for large-scale applications. These future directions will provide a broader and more balanced perspective on the scope and applicability of the present work.

## Figures and Tables

**Table 1:** Composition of experimental resins prepared with specified monomer percentages (% *w*/*w*).

Experimental Resins (ER)	Bis-GMA (%)	TEGDMA (%)	PET-GLY 1-DM (%)	PET-GLY 2-DM (%)
1	50	50		
2	60	40		
3	70	30		
4	40	50	10	
5	40	50		10
6	25	50		25

**Table 2:** Total mean values ± standard deviation (SD) calculated for degree of conversion (180 s light-curing, n = 5), total strain (n = 5), water sorption and solubility (n = 4). Different superscript lowercase letters indicate statistically significant differences within the same column data (*p* ≤ 0.05).

Experimental Resins (ER)	Final DC (%)	Total Strain (%)	Water Sorption (μg mm^−3^)	Water Solubility (μg mm^−3^)
1	59.56 ± 1.13 ^a^	7.96 ± 0.26 ^a^	36.72 ± 1.21 ^a^	1.86 ± 0.12 ^a^
2	55.26 ± 1.11 ^b^	6.79 ± 0.50 ^b^	26.13 ± 2.39 ^b^	3.23 ± 0.49 ^b^
3	53.46 ± 1.25 ^c,b^	6.09 ± 0.45 ^c^	23.85 ± 1.56 ^c,b^	3.62 ± 0.30 ^c,b^
4	62.5 ± 1.32 ^d^	8.13 ± 0.26 ^a^	33.36 ± 2.14 ^a,f^	1.71 ± 0.25 ^a^
5	64.72 ± 1.08 ^e,d^	8.4 ± 0.22 ^a,e^	32.19 ± 1.23 ^d,f^	1.53 ± 0.14 ^a^
6	69.54 ± 1.76 ^f^	8.85 ± 0.23 ^d,e^	29.09 ± 1.02 ^e,b^	1.28 ± 0.11 ^a^

**Table 3:** Total mean values ± standard deviation (SD) for flexural strength and elastic modulus (n = 5) before [25]. and after immersion in distilled water for 1 week. Different superscript lowercase letters indicate statistically significant differences within the same column data (*p* ≤ 0.05), while different superscript uppercase letters indicate statistically significant differences between these two properties before and after immersion (*p* ≤ 0.05).

Experimental Resins (ER)	FS Before Immersion (MPa)	EM Before Immersion (GPa)	FS After Immersion (MPa)	EM After Immersion (GPa)
1	93.89 ± 9.01 ^a A^	2.11 ± 0.38 ^a A^	65.53 ± 7.01 ^a A^	1.58 ± 0.27 ^a A^
2	115.90 ± 7.96 ^b B^	2.79 ± 0.39 ^a Β^	91.79 ± 6.4 ^b B^	2.33 ± 0.3 ^b Β^
3	129.21 ± 9.99 ^c,b C^	3.26 ± 0.44 ^b C^	102.5 ± 8.3 ^c,b C^	2.73 ± 0.36 ^c,b D^
4	82.28 ± 9.15 ^a,e D^	1.86 ± 0.21 ^a,d E^	60.26 ± 6.86 ^a,e D^	1.45 ± 0.24 ^a,d E^
5	78.54 ± 9.53 ^a,e E^	1.84 ± 0.29 ^a,d,e F^	58.14 ± 8.63 ^a,e E^	1.44 ± 0.25 ^a,d G^
6	60.24 ± 8.65 ^d F^	1.62 ± 0.28 ^c,d,e H^	46.33 ± 7.38 ^d,e G^	1.33 ± 0.13 ^a,d I^

**Table 4:** Total mean values ± standard deviation (SD) corresponding to the eluted content of BPA, TEGDMA and Bis-GMA from each experimental resin (n = 4). Different superscript lowercase letters indicate statistically significant differences within the same column data (*p* ≤ 0.05).

Experimental Resins (ER)	BPA (mg/mg_resin_)	TEGDMA (mg/mg_resin_)	Bis-GMA (mg/mg_resin_)
1	0.124 ± 0.021 ^a^	0.038 ± 0.002 ^a^	0.192 ± 0.017 ^a^
2	0.261 ± 0.016 ^b^	0.027 ± 0.001 ^b^	0.214 ± 0.025 ^a^
3	0.357 ± 0.045 ^c^	0.017 ± 0.002 ^c^	0.246 ± 0.030 ^a^
4	0.028 ± 0.004 ^d^	0.016 ± 0.001 ^d,c^	0.281 ± 0.019 ^b,e^
5	0.028 ± 0.004 ^e,d^	0.017 ± 0.001 ^e,d^	0.273 ± 0.045 ^c,e^
6	0.000 ± 0.000 ^f,d^	0.015 ± 0.002 ^f,c^	0.290 ± 0.032 ^d,e^

## Data Availability

The original contributions presented in this study are included in the article. Further inquiries can be directed to the corresponding author.

## Abbreviations

The following abbreviations are used in this manuscript:

Bis-GMA	2,2-Bis[p-(2′-hydroxy-3′-methacryloxypropoxy)phenylene]propane/bisphenol A glycidyl methacrylate
BPA	Bisphenol-A
CQ	Camphorquinone
DC	degree of conversion
DMAEMA	2-(dimethylamino)ethyl methacrylate
EM	elastic modulus
FS	flexural strength
GPC	gel permeation chromatography
LVDT	linear variable displacement transducer
PET	poly(ethylene terephthalate)
PET-GLY-DMs	dimethacrylated oligoesters
TEGDMA	triethylene glycol dimethacrylate
THF	Tetrahydrofuran
UDMA	urethane dimethacrylate
